# Views of health care users and providers: Solutions to improve the prevention of secondary health conditions among people with spinal cord injury, South Africa

**DOI:** 10.1038/s41394-022-00530-w

**Published:** 2022-07-19

**Authors:** Sonti Pilusa, Hellen Myezwa, Joanne Potterton

**Affiliations:** 1 Physiotherapy Department, School of Therapeutic Sciences, University of the Witwatersrand, Parktown, South Africa; 2 School of Therapeutic Sciences, University of the Witwatersrand, Parktown, South Africa

**Keywords:** Preventive medicine, Rehabilitation

## Abstract

**Study design:**

Explorative- qualitative study.

**Objective:**

This study explored solutions to improve the prevention of secondary health conditions in people with spinal cord injury.

**Setting:**

Rehabilitation hospital, South Africa.

**Methods:**

Face to face semi-structured interviews were conducted with 21 therapists and 17 people with spinal cord injury at a public rehabilitation hospital. All the interviews were transcribed verbatim. Content analysis was conducted on the transcripts to identify proposed solutions to improve the prevention of secondary health conditions.

**Results:**

The main theme that emerged was access to adequate health care. The categories linked to the main theme were: availability of health services, patient-centred care, strengthening rehabilitation care, access to resources and training health professionals.

**Conclusions:**

Access to adequate health is central to preventing and managing secondary health conditions. Care for people with spinal cord injury needs to be empowering and address rehabilitation care needs across the lifespan. The proposed solutions will inform the development of a prevention care model for secondary health conditions in people with spinal cord injury.

## Introduction

Health disparities still exist for people with chronic conditions such as spinal cord injury. People with spinal cord injury (SCI) have unmet care needs for health information and support to prevent secondary health conditions (SHCs), these include pain, pressure sores and urinary tract infections [1, 2]. Spinal cord injury care tends to be more reactive instead of proactively preventing SHCs occurrence and promoting health [3, 4]. Studies on SCI show a link between SHCs and increased readmission rates [5] and poor health and wellbeing [6]. The rising disability prevalence due to the burden of disease in South Africa increases the demand for comprehensive rehabilitation care and population-based public health programmes to minimise the occurrence of SHCs and prevent further disability [7, 8].

In order to strengthen SCI care and decrease repeated hospitalization due to SHCs, Dejong et al. [4] highlighted the importance of the health care system proactively preventing SHCs, focusing on patient-centered care, and applying systems thinking when developing prevention strategies. Systems thinking is a holistic and collaborative approach to solving problems by analysing the complexities and patterns to inform appropriate interventions for the identified problems [9]. Systems thinking encourages a shift from linear and reductionist approaches when solving health problems to multilevel strategies that address underlying influencing health factors [10]. An example of a model that incorporated multiple strategies to prevent SHCs is Rimmer’s conceptual model for identifying, preventing and managing secondary conditions in people with disabilities [11]. The model proposes four prevention strategies for SHCs: assistive devices, rehabilitation, health promotion and policies [11]. Evidence on the application of this model for people with SCI is limited.

Prevention care for SHCs requires a partnership between the patient and health providers [12]. The health providers’ role in preventing SHCs includes providing patient-oriented care, empowering individuals with SCI with information on SCI and SHCs, self-management skills, and actively engaging patients in planning and decision-making [12, 13]. The patient’s role is to take responsibility for their health, self-manage, and seek help when needed [14]. Building this partnership has its challenges in understanding each stakeholder’s role and setting patient care goals [12]. However, to enhance inclusive health and develop a responsive prevention care model, we need to listen to both the service users’ and care providers’ views on health and ways to promote well-being. Thus, this study explored solutions to improve the prevention of SHCs in people with SCI.

## Method

### Study design

An explorative qualitative approach was used to understand the participants’ views on solutions to improve the prevention of SHCs. This study is part of a bigger study on the factors influencing the prevention of SHCs in people with SCI [15]. The study involved identifying the prevalence of SHCs and exploring SHCs’ preventing strategies and factors that influence the prevention of SHCs to inform the development of a prevention model of care.

### Study context

The study setting was a public rehabilitation hospital based in Gauteng province, the economic hub and a populous province in South Africa. Tshwane rehabilitation hospital offers in-patient rehabilitation and monthly outpatient medical services for patients with physical disabilities. Due to the lack of rehabilitation hospitals in the country, people with SCI travel from other provinces to access rehabilitation services.

The majority of the South African population (84%) use the public health care system that is overburdened and short-staffed [16]. Accessing rehabilitation care is also limited for people with disabilities due to less priority on long-term care [7], attitudinal, geographical and monetary healthcare access barriers [17]. Although people with disabilities have access to social grants, the grant is still not sufficient to cover costs related to living with a disability. There is no national data on the burden of SCI and the only available SCI epidemiological data is from one province (Cape Town).

### Participants

We used purposive sampling to recruit the participants. The participants included individuals with SCI using outpatient medical services at the rehabilitation hospital. All the participants had to be 18 years or older to participate in the study. We only included people with SCI and therapists who gave informed consent. People with SCI were included irrespective of sex and SCI injury profile (lesion level, type of injury and duration of the injury).

All public hospitals use an income classification to classify patients’ income and to determine the subsidy for healthcare costs:

H0—Social grant, pensioners and unemployed: full subsidy.

H1—0 to 70 000 per annum (Single income) and 0-100 000 per annum (Household income): partial subsidy.

H2—70001 to 250 000 (Single income)/100 001 to 350 000 per annum (Household income): partial subsidy.

H3—>250 000 per annum-(Single income)/>350 000 (Household income): partial subsidy.

Road Accident Fund — eligible patients who were involved in a pedestrian or motor vehicle accident: no subsidy.

### Data collection

The researchers developed an interview guide and piloted it to ensure the clarity of the questions. The topics covered in the interview guide included common SHCs experienced by people with SCI, prevention strategies for SHCs, factors influencing the prevention of SHCs and solutions to improve the prevention of SHCs. The principal author explained the study to eligible participants, obtained informed consent, and audio recorded the interviews. Face to face interviews were conducted at the hospital in the participant’s language of choice. On average, the interviews lasted 45 min. Data collection was from August 2018 to July 2019 and was conducted until data saturation was reached.

### Data analysis

All the interviews were transcribed verbatim. The principal author read all the transcripts to ensure correct transcription and a sense of narratives. Qualitative data analysis software (MAXQDA version 2018.1) was used to manage and analyze the data following content analysis steps outlined by Erlingsson and Brysiewicz [18]. Using a coding framework developed collectively by the authors, inductive and deductive analysis was conducted, the text was transformed into codes and categories [18]. Debriefing sessions were conducted throughout the research process. The participants’ names were not used to ensure anonymity, and the quotes were labelled (SCI, 1, Therapist 1).

### Ethical considerations

Data was collected after approval from the Human Research Ethics Committee of the University of the Witwatersrand (M170938), South African National Health Research Database (reference GP201712036) and the study site was granted. Only participants who gave informed consent and permission to record interviews were included in the study.

We certify that all applicable institutional and governmental regulations concerning the ethical use of human volunteers were followed during the course of this research.

## Results

The results are presented in two sections, Part A: demographic profile of the participants and Part B: Emerged theme and categories related to the study aim.

### Part A: Demographic profile of the participants

We interviewed thirty-eight participants (21 therapists; 17 patients with SCI). Tables 1, 2, and 3 provides an overview of the participants’ demographic data and SCI profile. The majority of the participants with SCI were male and had traumatic SCI. The majority of the therapists were occupational therapists and physiotherapists.

**Table 1:** Demographic profile of the participants.

Participants with spinal cord injury *n* = 17 (%)	
**Age in years**	
Mean (SD), Range (years)	44.5 (13.1), 27–72
**Gender**	
Male	14 (82.4)
Female	3 (17.6)
**Employed,** ***n*** **(%)**	
Yes	5 (29.4)
No	12 (70.6)
**Ethnicity**	
Black	11 (64.7)
White	6 (32.3)
**Educational level**	
School <7 years	2 (11.8)
Secondary >7 years	12 (70.6)
Tertiary (Diploma and University qualifications)	3(17.6)
**Patient income classification**	
H0 - Social grant, pensioners and unemployed, full subsidy	9 (52.9)
H1 (0–70 000 per annum (Single income) and 0–100,000 per annum (Household income)	0
H2 (70001– 250,000- Single income/100,001–350,000 per annum-Household income)	0
H3 ( > 250,000 per annum-Single income/> 350,000 Household income)	1 (5.9)
Road Accident Fund	7 (41.2)
**Assistive device**	
Wheelchair	14 (82.3)
Walking aid	2 (11.8))
None	1 (5.9)
**Geographic location**	
Urban	12 (70.6)
Rural	5 (29.4)
**Distance patients travel to the hospital**	
Mean(km) Range (km)	Mean 40 km
	Range 3–145 km

**Table 2:** SCI profile.

Time since injury	
Mean (SD), Range (years)	9 (7.1), 1–30
**Cause of injury**	
Trauma	14 (82.4)
Non-traumatic	3 (17.6)
**Type of spinal cord injury**	
Paraplegia	14 (82.4)
Tetraplegia	3 (17.6)
**Completeness of the injury**	
Incomplete	4 (23.5)
Complete	13 (76.5)
**Level of the injury**	
**C1-C4**	2 (11.8)
**C5-T1**	1 (5.9)
**T2-T6**	3 (17.6)
**T7-T12**	9 (52.9)
**L1-L5**	2 (11.8)

**Table 3:** Demographic data for the therapists.

Therapists *n* = 21 (%)	
**Age (years)**	
Mean (SD)	31.5 (8.3)
Range	22–54
**Gender,** ***n*** **(%)**	
Female	17 (81)
Male	5 (19)
**Professions,** ***n*** **(%)**	
Occupational therapy	7 (33.3)
Physiotherapy	6 (28.5)
Social worker	1 (4.8)
Psychologist	1 (4.8)
Speech therapist	2 (9.5)
Dietician	3 (14.3)
Occupational therapist assistant	1 (4.8)
**Work experience (years)**	
Mean (SD)	8.7 (8.5)
Range	1–28
**Ethnicity**	
Black	12 (57)
White	9 (43)

All of the participants with SCI had at least one SHCs, and pain was the most prevalent SHCs. Table 4 illustrates the the range fo SHCs experienced by the participants with SCI.

### PART B: Theme and categories related to the study aim

The main theme that emerged was “Access to adequate health care”. The five categories linked to the main theme were:

Availability of health care

Patient-centred care

Strengthen rehabilitation care

Access to resources

Train health professionals.

### Availability of health care

Participants highlighted the need for health care services closer to where they live for pressure sore management care, weekly check-up and follow-up care.

*“The government can play a role in building a pressure sore clinic in the community where we could go weekly for check-ups”* (SCI 3)

*“We need clinics nearby with a rehab facility so that the patients can do check-ups and follow-ups there. One patient comes from Mpumalanga once a month because no clinic helps her there. So, just accessibility, care closer to the patient”* (Therapist 15)

*“I would prefer if there were physios in the townships as well, where we could go and train during the week just like we do here at the rehabilitation hospital"* (SCI 1)

### Patient-centered care

Participants with SCI emphasized the importance of patient-centered care, and the sub-categories include listening to patients, health education on SHCs and prevention, and patient support system.

Participants with SCI wanted health professionals to listen to them, involve them in decision-making, and care planning.

*“What I am fighting for most is that they will listen to me as a patient. It does not matter how much you have studied, listen to me and what I am saying instead of acting like they know”* (SCI 6)

*“There is only one solution. Listen to people. …we are not the same… But, if only they could listen so that they can know the challenges patients have. How they feel about it. The big thing is, you have to ask the patient….ask the patient about the injury”* (SCI7)

*“I am not scared to tell them that yes, you studied but not what I am suffering from, this person has never been in my shoes, and the doctor will never know the pain I am in and how I feel. Just listen to me, as I said the neurologists from hospital X, I used to say ..how about you book me for this test and let’s see what you will find and that leads to other tests”* (SCI 6)

Most participants mentioned that education for SCI and family on SHCs, SHCs preventative care, self-management and information on assistive devices was necessary to help in preventive care for SHCs.

*“The main issue is pressure sores, it bothers people who use wheelchairs, I wish they could teach us more about prevention, and I wish they could explain well how pressure sore develop*” (SCI 5)

*“People need to be properly taught how to manage secondary conditions… and when you experience a problem, do not just leave it and think it will go away, make sure that you take advice, go to speak to someone, and even get a second opinion if needs be”* (SCI 11)

*“Educate the family and talk to them about the possible risks so they can notice the signs and symptoms in mental health”* (Therapist 7)

Participants commented on the importance of a support system for people with SCI to help them manage their disability, reinforce what they learned on SHCs prevention, and share their experiences. The support could be from peers with disabilities and community support groups.

*“We can have support groups where people with spinal cord injury can go for support, debriefing and reinforcement of preventative measures”* (Therapist 13)

*“We need (non-government organisations) NGOs to go to during weekends where we can meet and talk. You can have a normal person as a friend, but it is better if it is someone who is going through more or less the same thing as you are. Maybe they can share something they are going through. It would encourage me to be like them instead of stressing about all these other things*” (SCI 12)

*“What can help is if there are places where persons with SCI can get information from, phone and say, listen, I have this problem, and they can say, okay, we have a network of people, we can get you to contact the people doing this and that…. adapting your house or whatever is needed…or we can give a number for a doctor or something that can explain pressure sores.”* (SCI 15)

### Strengthen rehabilitation care

Participants stressed the importance of strengthening rehabilitation care by ensuring that it is needs-based, supports the transition process, and facilitates continuity of care when they are home. In addition, patients’ needs must inform rehabilitation care, and rehabilitation must start early to enhance ownership of personal health.

*“They should visit the patients’ homes before they discharge them….so they can know that this person’s bed is this high, he baths in a bucket… we cannot be trained the same way as someone who uses a bathroom tub”* (SCI 12)

*“You rehab your patients, you do your toilet transfer where there is something to hold on, but when you get home, it is a different thing, so yes, we are doing our best, but we are not rehabilitating our patients to go function in their settings, that is one of the gaps that I feel that we need to close”* (Therapist 2)

The participants discussed that transition from hospital to home needs to be managed carefully to ensure patients are able to cope and family could be involved in the process.

*“I think the transition from the rehabilitation hospital to the community is important because the hospital is a controlled environment. When the patients with spinal cord injury go back home, it is a completely different environment of which they tend not to cope most of them”* (Therapist 13)

*“We must involve the family as much as possible to ensure successful transition from rehab to home”* (Therapist 11)

The participants stressed the importance of continuity of care through regular patient follow-up, home visits, and outreach services to ensure patients progressed well and managed to prevent SHCs.

Health professionals, community-based workers and peers with disabilities could play a vital role in follow-up care post-discharge.

*“A multidisciplinary team must see patients… drive to their homes… check on them just to check the environment and also ensure they are fine”* (Therapist13)

*“At a healthcare level we need to improve on home visits on a regular bases, just to follow up, catch problems before…they are major….”* (Therapist 10)

Lastly, linked to rehabilitation care, participants discussed reintegrating people with SCI back to work and leisure to give them a sense of meaning and help them prevent SHCs.

*“Invest money in getting sheltered employment for these people because that is important. If I used to wake up every day and have a life and suddenly no one wants to employ you, that will depress me as well”* (Therapist 10)

*“Re-integration is not just about going back to your family. It is going back out there, going back to things you used to do, leisure, if people are not working, they just sit at home …. They should do things that will encourage them to have something to live for, and I think that is very important”* (Therapist 10)

*“And that is one thing I can say it is very difficult, is to get a proper sports center where we can go and do sports… I am talking about getting involved with sports for people with disabilities”* (SCI 17)

### Access to resources

Participants identified basic and essential resources for people with SCI to enhance the prevention of SHCs.

### Medication and assistive devices

Participants mentioned that people with SCI needed adequate supply of medication, pressure sore dressings, consumables for bladder management and nutritional supplements to support the prevention of SHCs.

*“Give us enough medication. ….It is painful being unable to sleep because of pain, and you have to wait for three months to get more medication”* (SCI 5)

*“We need adequate drugs, dressings, (uhm).. also if we are giving patients uhm…., catheters …. using a single-use hydrophilic catheter decreases uhm…. urinary tract infections, but we do not have those (single-use hydrophilic catheters), so if we could give single-use hydrophilic catheters to patients, it will help to prevent UTI”*. (Therapist 16)

Participants spoke about the importance of assistive devices and that they must be easily accessible, appropriate, and of acceptable quality.

*“We also need access as well to other types of cushions, or wheelchairs because we only have the standardized ones (wheelchairs) which are not always good for all patients. This could also help in preventing pressure sores”* (Therapist 1)

***“****A patient should be able to get the necessary wheelchair that they need at the right time, not waiting ten years to get a new chair”* (Therapist 15)

### Health professionals

The participants highlighted the need for more health professional staff to ensure continuity of care and patient-centered care.

*“I would start with ensuring that there is adequate healthcare staff because I think the nurses develop bad attitude because they are also fatigued”* (Therapist 21)

*“Invest in increasing the number of employees in our hospital so that people do not complain over being short-staffed”* (Therapist 10)

### Other basic resources

Participants suggested a disability-friendly transport system that can transport people with wheelchairs to better access health services. For example, a participant with SCI said:

*“There should be public transport vehicles that can load wheelchairs… with enough space for people and their wheelchairs. Sometimes we wait long for taxis – most of them are not willing to transport us with our wheelchairs… also some people are not able to transfer themselves into the taxis”* (SCI 1)

*“We need to make sure there is easier transport for them (people with disabilities) so that they can get to the clinics and the workplaces”* (Therapist 20)

Participants with SCI mentioned access to safe drinking water and adequate toilet facilities inside the house to make daily living activities easier.

*“What could make me happy is that the house I live in should have a tap for water inside”* (SCI 14)

*“If I could get a toilet inside the house, managing my bowel will be easier “*(SCI 12)

### Train health professionals

Participants noted a gap in the health professionals’ knowledge on SCI and SHC prevention. They suggested that topics including disability literacy, SCI care and treating pressure sores could be included in the training of health professionals.

*“Teach health professionals on how to work with people with spinal injuries, especially when they are in the hospital”* (SCI15)

*“Teach the NGOs, community-based nurses and home-based carers how to treat pressure sores” (*SCI 1)

“*I think that maybe the nursing staff should be trained on dressing and treating pressure sores because sometimes some of them treat it, but they do not do it the same”* (SCI 2)

*“The training for health professionals should be in such a way that if someone comes with a disability, they know at least what the condition is and how they can help before they transfer to the hospital. But the problem is that I do not think training is that deep, especially when it comes to SCI…”* (Therapist 5)

## Discussion

This study explored solutions to improve the prevention of SHCs in people with SCI. From the analysis, the key theme that emerged was “access to adequate health care” with five categories: availability of health care, patient-centred care, strengthening rehabilitation care, access to resources and training health professionals. Adequate access to health care can be described as one’s ability to identify personal health care needs, seek and use health services to address health needs entirely [16]. The dimensions of healthcare access include acceptability, availability, accommodation, affordability and appropriateness [19]. Access to health care including rehabilitation services, close to where people live, is a human right that people with disabilities have not fully realised [17, 20].

Research shows that access to health care for people with disabilities is limited [17, 21]. There is a shortage of rehabilitation services at a primary care level [22], unmet assistive devices needs, and a lack of home-based care in South Africa [17]. However, the unavailability of rehabilitation services could be due to the shortage of rehabilitation therapists [23]. The ratio of rehabilitation therapists was 7.25/100,000 population in 2019, which translates to 3.10 physiotherapists, 2.64 occupational therapists and 1.51 speech therapists/ 100 000 [23]. The lack of rehabilitation services in the community forces people with disabilities to travel long distances to access healthcare. Nteta et al. [24] reported that most people based in an urban setting travelled less than 5 km to access a primary health facility, either using a taxi or walking. Although this study reported good access to primary health facilities, none of the study participants reported having a disability. The experiences of people with disabilities are very different. Even if health services are available, getting to the health care facility is not accessible, due to lack of disability-friendly transport, high transport costs, architectural and terrain barriers [17, 21, 25]. The study findings point to the need to strengthen access to health care for people with disabilities. Rehabilitation therapists and community-based workers can conduct community-based outreach services and home-based care to bridge this gap.

Spinal cord injury is a chronic condition with complex and varying needs due to secondary complications; thus, a patient-centred care approach is needed [26]. People with SCI need holistic care and assistance to live well with a chronic condition. However, the health system is medically oriented, thus ignoring the long-term needs of living with a chronic disease [22]. People with chronic conditions and multi-morbidity are treated in silos with repeated assessments done at different points of care, that do not necessarily lead to better health outcomes [27]. Systemic health system challenges such as lack of resources, shortage of human resources, curative focus to care and fragmented care limit patient-centred care [28, 29].

Patient-centred care is an enabling approach that puts the patient at the centre of care by involving them in decision making, planning, recognising the patients’ role and their context in personal well-being [30]. Patient-centred care communicates respect and value to the patients by listening to their experiences of illness and health goals [26]. To strengthen patient-centred care, we need to train health professionals to render holistic patient-centred care across the continuum[31]. Undergraduate and continuous in-service training can include patient-centred care, a biopsychosocial approach to health, the importance of health maintenance and preventative care. [10, 31]. Secondly, organisational changes to support patient-centred care are recommended, such as hiring more health professionals, encouraging collaborative care and patient follow-up [26, 31].

Participants highlighted the importance of educating people with SCI on SHCs and prevention care to address the unmet need for health information [2]. Health literacy is a vital tool in Sustainable Development Goal no.3 to promote health and behaviour change [32]. Increasing awareness on SCI, self-management and health maintenance is vital for chronic management and patient-centred care [33, 34]. Factors such as age, beliefs about risks [1], educational levels, previous history of SHCs, marital status and access to online material and technology influence access to health information and must be taken into account when educating people with SCI [35]. An untapped resource for health information in South Africa is peer education and telehealth [4]. Future research can explore the role of telehealth and peer mentors in increasing health awareness for people with SCI.

Social support potentially increases access to health services, encourages self-management [36] and promotes the prevention of SHCs [3]. Involving the family in care, planning and decision making can strengthen patient-centred care and ensure continuity of care at home [22]. The second source of support is health professionals who can provide continuity of care through home visits, outreach services and regular check-up sessions [12, 37]. Unfortunately, health professionals lack of knowledge on SCI and SHCs [38]. Shortage of health professionals [39], and lack of transport for home visits [22] affect the level of support given to patients post-discharge. A new cadre of community-based rehabilitation care workers was introduced to strengthen primary health care and support community-based rehabilitation in South Africa [40]. However, there are challenges in incorporating this group into the broader health care service [8]. Strengthening partnerships with rehabilitation care workers can improve skills transfer, continuity of care, and enhance patient support.

The proposed solutions align with the strategies to prevent SHCs in Rimmer’s conceptual model [11]. The solutions cover broader aspects of the patient centred care approach in supporting prevention of SHCs, focusing on the healthcare user, healthcare provider and the healthcare organisation. These findings confirm the importance of systems thinking when strengthening prevention care in people with SCI. Systems-wide multilevel interventions can better respond to complex health problems.

## Conclusion

The proposed solutions to improve the prevention of SHCs revolve around access to adequate health care. The study findings support the call to integrate rehabilitation at all levels of care [7, 17]. This will improve access to care, including preventive care, planning and resource allocation for assistive devices, medication and human resources.

### Limitations

The study findings apply to the study setting and cannot be generalised to other contexts considering the qualitative study design. We had few female participants with SCI. Although SCI tends to affect more males than females, understanding women’s views would have been beneficial given the socioeconomic inequalities women experience in South Africa that can limit access to health. Community-based studies targeting women with SCI are recommended to bridge access barriers. The overall aim of the study was to gain a broad understanding of personal and environmental factors influencing the prevention of SHCs and to identify solutions to improve the prevention care. Thus, adult age group were included in the study. The majority of the participants fall in the working-age group except for three participants who were already pensioners. The selection of participants based on age should be considered in future studies to ensure need -based care programmes. The majority of the participants with SCI were unemployed and depended on the social grant. Recruiting patients at the rehabilitation hospital could have limited the inclusion of patients with SCI who cannot afford transport to access the rehabilitation hospital

### Recommendations

#### Research

Future research can implement the proposed solutions and assess their effectiveness. Apply system thinking tools in the design and evaluation of prevention interventions.

Future research can explore the use of telehealth and peer mentors in increasing health awareness for people with SCI.

#### Practice

Health professionals must educate their patients and build self-management skills.

#### Education

Train health professionals in both undergraduate programmes and continuous professional development workshops on patient-centred care, the biopsychosocial approach to health, the importance of health maintenance and preventative care. Table 4

**Table 4:** Outline of the secondary health conditions *n* = 17 [6].

Type of secondary health condition		*n* (%)
Pain		16 (94)
Bladder problems	Incontinence	16 (94)
	Urinary tract infection	4 (24)
	Spastic bladder	2 (12)
	Kidney stones	1 (6)
Bowel problems	Incontinence	11 (65)
	Constipation	6 (35)
	Rectal prolapse	1 (6)
	Bloating	1 (6)
Psychological problems (depression, worry, stress)		12 (71)
Pressure sores		10 (59)
Spasms		9 (53)
Contractures		3 (18)
Injuries	Burns	9 (53)
	Fractures	4 (24)
	Falls	8 (47)
Sexual issues		7 (41)
Sleeping disturbances		6 (35)
Fatigue		4 (24)
Skeletal problems	Osteoporosis	2 (12)
	Arthritis	1 (6)
	Myositis ossificans	1 (6)
Respiratory problems		1 (6)

## Supplementary information

Appendix 1

Appendix 2

## Data Availability

All data generated or analysed during this study are included in this published article, and its supplementary information file is available from the corresponding author on reasonable request.
