# Supplementary material for: Views of health care users and providers: Solutions to improve the prevention of secondary health conditions among people with spinal cord injury, South Africa
Source: Spinal Cord Ser Cases. 2022 Jul 19;8:67. doi: 10.1038/s41394-022-00530-w (PMC9296448; doi:10.1038/s41394-022-00530-w)
Supplement: Supplementary file 1 — Appendix 1 [file 41394_2022_530_MOESM1_ESM.docx]

**Appendix**

**Interview guide**

| **Introduction** | Good day…can you tell me about yourself |
| --- | --- |
| **Theme 1: Disability** | Can you tell me about your disability? Can you tell me about your experience living with a disability?  Probe: how and when the disability occurred? |
| **Theme 2: Secondary health conditions experienced by** People with spinal cord injury | People with spinal cord injury sometimes experience secondary health conditions/ secondary complications….  What is your understanding of secondary complications commonly experienced by people with spinal cord injury?  Can you tell me about any Secondary complications related to the spinal cord injury  Which secondary complications have you experienced?  Why do they happen if you were rehabilitated? (Underlying risk factors?)  Probe: why did you experience these conditions? |
| **Theme 3: Prevention Strategies of secondary complications** | How do you prevent or manage secondary complications in your life?  Probe: “other people prevent secondary complications e.g. pressure sores by doing regular pressure relief” can you share more on how you prevent secondary complications from occurring in your life.  Can you share more on how you manage/d them when you had secondary complications |
| **Theme 4: Factors (personal and environmental) influencing prevention of secondary complications** | Can you share a typical scenario where there were factors that made prevention of secondary complications manageable or easy? (Facilitators)  Probe: what made it easier for you to prevent them from occurring?  Probe: Why?  Can you share a typical scenario where there were factors that made prevention of secondary health conditions difficult? (barriers) What made it harder for you to prevent the secondary complications  Probe: Why? Can you share the challenges you have experienced in preventing or managing secondary health conditions  Probe: Factors from the rehabilitation hospital?  Probe: Factors from the family? Community? |
| **Suggestions** | If you had the power to change things … Which solutions or strategies might assist the prevention of secondary complications/secondary health conditions?  Any THOUGHTS on how prevention care for secondary complications can be improved in people with spinal cord injury. |
