# Supplementary material for: Views of health care users and providers: Solutions to improve the prevention of secondary health conditions among people with spinal cord injury, South Africa
Source: Spinal Cord Ser Cases. 2022 Jul 19;8:67. doi: 10.1038/s41394-022-00530-w (PMC9296448; doi:10.1038/s41394-022-00530-w)
Supplement: Supplementary file 2 — Appendix 2 [file 41394_2022_530_MOESM2_ESM.docx]

**Appendix 2: Semi structured interview guide for the health therapists**

**Section 1: Demographic data sheet for key informants**

| **Gender** |  |
| --- | --- |
| **Age** |  |
| **Level of education** |  |
| **Occupation** |  |

**Interview guide**

| **Theme 1: key informant role** | Can you tell me a little bit about your role in this rehabilitation  Can you tell me a little bit about your care for the patient with spinal cord injuries? |
| --- | --- |
| **Theme 2: prevention of secondary health conditions** | Tell me about secondary health conditions/secondary complications commonly experienced by people with spinal cord injury.  Can you share how these conditions are prevented?  Probe: How do you assist patients to prevent secondary health conditions? |
| **Theme 3: Factors** **influencing prevention of secondary health conditions** | What factors across the continuum of care from hospital to home setting influence the prevention of secondary complications?  What are some of the things that make prevention of secondary health conditions hard for patients? (barriers)  Why?  What are some of the things that make the prevention of secondary health conditions manageable or easy? (Facilitators) |
| **Suggestions** | What are some of the strategies and solutions that might assist people with SCI to prevent of secondary health conditions?  Do you have any suggestions on how prevention (care) of secondary conditions can be improved? |
